# Canadian Lung Cancer Relative Risk from Radon Exposure for Short Periods in Childhood Compared to a Lifetime

**DOI:** 10.3390/ijerph10051916

**Published:** 2013-05-08

**Authors:** Jing Chen

**Affiliations:** Radiation Protection Bureau, Health Canada, 2720 Riverside Drive, Ottawa, ON K1A 0K9, Canada; E-Mail: jing.chen@hc-sc.gc.ca; Tel.: +1-613-941-5191; Fax: +1-613-960-5604.

**Keywords:** radon-222, radon, indoor exposure, lung cancer risk

## Abstract

Long-term exposure to elevated indoor radon concentrations has been determined to be the second leading cause of lung cancer in adults after tobacco smoking. With the establishment of a National Radon Program in Canada in 2007 thousands of homes across the country have been tested for radon. Although the vast majority of people are exposed to low or moderate radon concentrations; from time to time; there are homes found with very high concentrations of radon. Among those living in homes with very high radon concentrations, it is typically parents of young children that demonstrate a great deal of concern. They want to know the equivalent risk in terms of the lifetime relative risk of developing lung cancer when a child has lived in a home with high radon for a few years. An answer to this question of risk equivalency is proposed in this paper. The results demonstrate clearly that the higher the radon concentration; the sooner remedial measures should be undertaken; as recommended by Health Canada in the Canadian radon guideline.

## 1. Introduction

Radon is a radioactive gas produced by the decay of natural uranium in rocks and soils throughout the Earth’s crust. A certain fraction of the radon escapes into the air. Outdoors, radon is quickly diluted by atmospheric mixing and is of no further concern. However, in confined spaces such as residential homes, radon can accumulate to harmful levels. Long-term exposure to elevated indoor radon concentrations has been determined to be the second leading cause of lung cancer in adults after tobacco smoking [1].

Recent scientific studies [2,3] provided strong evidence to link an increased risk of developing lung cancer to levels of radon found in homes. These studies prompted the Government of Canada to collaborate with provincial and territorial governments to review the Canadian radon guideline. Following a broad stakeholder and public consultation, in June 2007 the Canadian guideline was lowered from 800 to 200 Bq/m^3^ [4]. Compared to baseline lung cancer rates, the relative risk for developing lung cancer for a non-smoker is doubled for a lifetime exposure at 200 Bq/m^3^ and was thought to represent a risk level at which non-smokers would be willing to take remedial action [4,5].

Following the revision to the guideline, a national radon education and awareness program officially began in 2008. The program is focused on raising awareness about radon, the potential health risks from exposure and encouraging Canadians to test their homes and to reduce radon levels, if necessary. Since the program began thousands of Canadians have tested the radon levels in their homes. Although the vast majority of people are exposed to low or moderate indoor radon concentrations, on occasion homes with very high concentrations of radon are found. Among those living in homes tested with high radon concentrations, parents of young children are often the most concerned. They want to know the equivalent risk in terms of the lifetime relative risk of developing lung cancer for childhood radon exposure. They want to understand the lifetime risk for their children and what the equivalent lifetime radon exposure is when their children have lived in high radon homes for a few years? An answer to this question of risk equivalency is proposed here.

### 1.1. Lifetime Relative Risk of Radon Induced Lung Cancer

Lifetime risk is the risk of developing a disease during one’s lifetime. Childhood exposure is the exposure received before the age of 16 in this study. Based on Canadian age-specific rates for overall and lung cancer mortalities (1996–2000) and the Canadian smoking prevalence data in 2002, the estimated baseline lifetime risk of developing lung cancer is 8.1% for males and 4.7% for females [5]. For Canadian non-smokers, the estimated baseline lifetime risk of developing lung cancer is 0.92% for males and 1.1% for females [5].

The calculations for lifetime risk of radon induced lung cancer are based on the risk model developed by U.S. Environmental Protection Agency (EPA) [6]. The EPA radon risk model is a modified model of the National Research Council, Biological Effects of Ionizing Radiations (BEIR) VI committee [7]. The EPA model is a single model which gives risk values midway between those obtained from the two BEIR VI preferred models. Although the models are based on epidemiological studies of adult male miners, they are widely accepted in lifetime risk assessment for indoor radon exposure. In summary, the mathematical form of the EPA model for the excess relative risk (*ERR*) at a given age, *e_a_*, is described as:

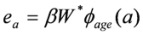
(1)
where *a* is age in years. The parameter *β* (=0.0634) represents the slope of the exposure-risk relationship. The parameter *Φ_age_(a)* describes the decrease of excess relative risk with increasing age. The continuous function of *Φ_age_(a)* given by the EPA is used in the current calculations. For a given radon concentration, the total exposure, W^*^, can be calculated as the weighted summation of three time-since-exposure windows, namely 5–14, 15–24, and 25 or more years before age a. Exposure in the last 5 years is not biologically relevant to cancer risk:

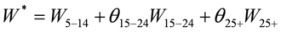
(2)
where *W_5–14_* is the exposure incurred between 5 and 14 years before age *a*; *W_15–24_* the exposure incurred between 15 and 24 years before age *a*; and *W_25+_* the exposure incurred 25 years or more before age *a*. In addition, *θ_15–24_* (=0.78) and *θ_15+_* (=0.51) represent the weights of the 15–24 and ≥25 time-since-exposure windows. 

The risk model, described with Equations (1) and (2) above, applies to all ages at exposure, from newborn to 110 years of age. It is well known that almost all risk estimates, including the BEIR VI models used here, are dependent on the modeling framework used for the data analysis. The BEIR VI committee used relative risk models to analyse the data of 11 miner cohorts [7]. The BEIR VI committee identified 13 sources of uncertainty in its estimates of risks from indoor radon. These were divided into two categories: (1) uncertainties in the parameter estimates for the exposure-response model derived from the miner data and (2) uncertainties in specifying the form of the model and in its application to the general population in residential settings. The risk model given above only provides the centre estimates from the best fits to the miner data. Essentially all the data on childhood exposures to radon were obtained from the Chinese tin miner cohort (one of the 11 miner cohorts), and those data are relatively sparse. Therefore, the uncertainty in risks associated with childhood exposures must be regarded as substantially higher than for adult exposures. Before more data become available for childhood radon exposure, no adjustment is made to the risk model for children’s sensitivity to radon. However, we should be aware of large uncertainties associated with the risk estimate, especially for childhood exposure.

The formulae for the calculation of lifetime relative risk of lung cancer are described in the BEIR IV report [8]. To calculate the probability of dying of lung cancer, suppose *q_a_* is the probability of surviving year *a* when all causes are acting on surviving through year *a−1*; 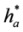 is the mortality rate due to all causes; and *h_a_* is the lung cancer mortality rate at year or age *a*. Then, the probability surviving year *a* is *q_a_ = exp(−* 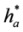 *)*, and the probability of death in year *a* is *1 − q_a_*. The probability of surviving up to age *a* is the product of surviving each prior year: 
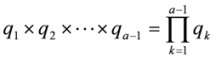
 . The probability of surviving up to age *a* and dying at age *a* is then 
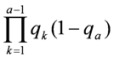
 . Multiplying by the proportion of the cause of lung cancer among all causes, the probability of surviving up to age *a* and dying of lung cancer at age *a* is 
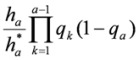
 . The lifetime probability of lung cancer mortality is the summation over all years where maximum life is assumed to be 110 years:

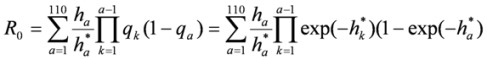
(3)


The additional risk of lung cancer due to exposure to radon is incorporated into the risk calculation of Equation (3) through the age-specific lung cancer mortality rates. The lung cancer mortality rate for an exposed individual is 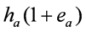 , and the overall mortality rate is 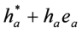 , where *e_a_* is the excess relative risk at age *a* as given in Equation (1). In the presence of radon exposure, the probability of surviving year *a* becomes 
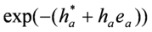
 , and the probability of death at age *a* is 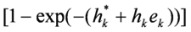 . The probability of surviving up to age *a* is the product of surviving each prior year from *1* to *a−1*, *i.e.* 
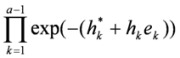
 . The probability of surviving up to age *a* and dying at age *a* is then 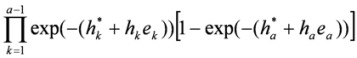 . Corresponding to Equation (3), multiplying by the proportion of lung cancer among all causes 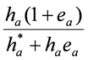 and summing over the lifespan of 110 years, the lifetime probability of lung cancer for an individual with excess risk profile *e_1_*, *e_2_*, *…*, *e_110_* is:

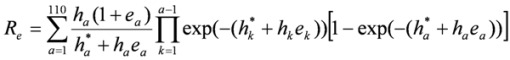
(4)
where *R_e_* is the lifetime risk of lung-cancer under a given radon exposure profile. 

The computation of lifetime risk depends on the choice of the background age-specific lung-cancer and overall mortality rates, *h_a _* and 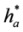. As in the previous publication [5], this study uses Canadian age-specific mortality rates averaged over five years from 1996 to 2000 [9].

Lifetime relative risk (LRR) is defined as 
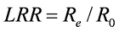
 , where *R_e_* is the lifetime risk of lung-cancer under a given exposure pattern (Equation (4)) and *R_0_* is the baseline risk (Equation (3)), *i.e.*, the lifetime risk for lung cancer of all causes including exposed to background radon level (the outdoor radon level~10 Bq/m^3^). As mentioned above, *R_0_* is estimated to be 8.1% for Canadian males and 4.7% for Canadian females. The *LRR* describes the proportional increment in lung-cancer risk posed by indoor radon exposure beyond the background level of exposures from outdoor air. 

**Figure 1:**
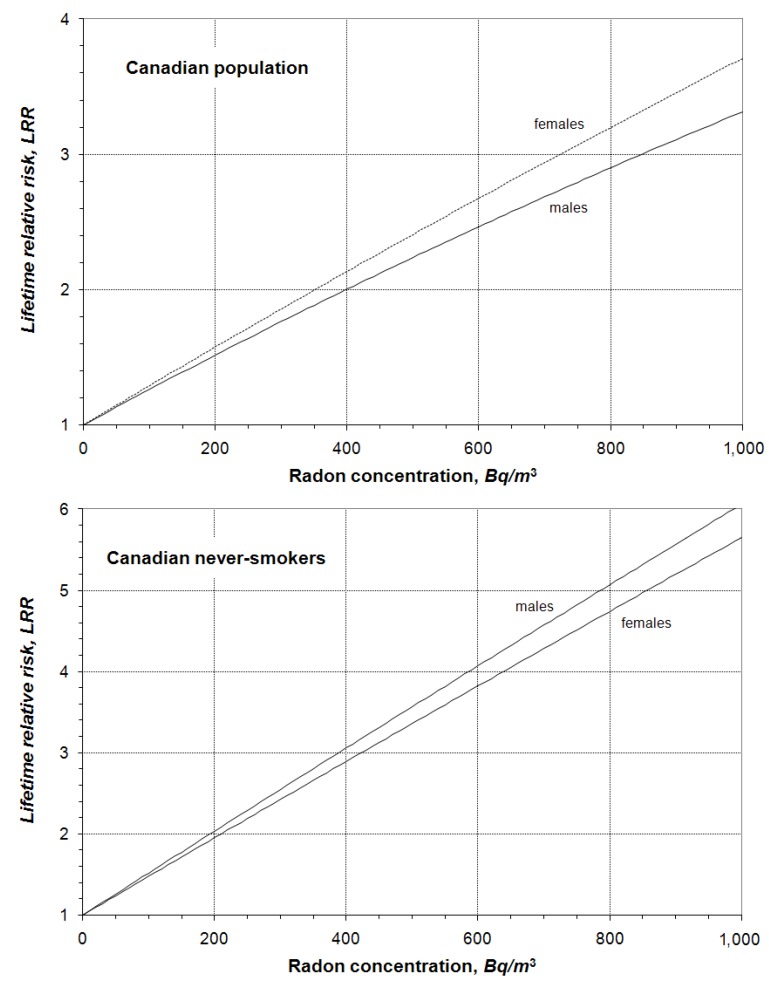
Lifetime relative risks of radon induced lung cancer for lifetime exposure at given radon concentration (upper panel for Canadian population—A mixture of smokers and non-smokers; lower panel for male and female never-smokers).

**Figure 2:**
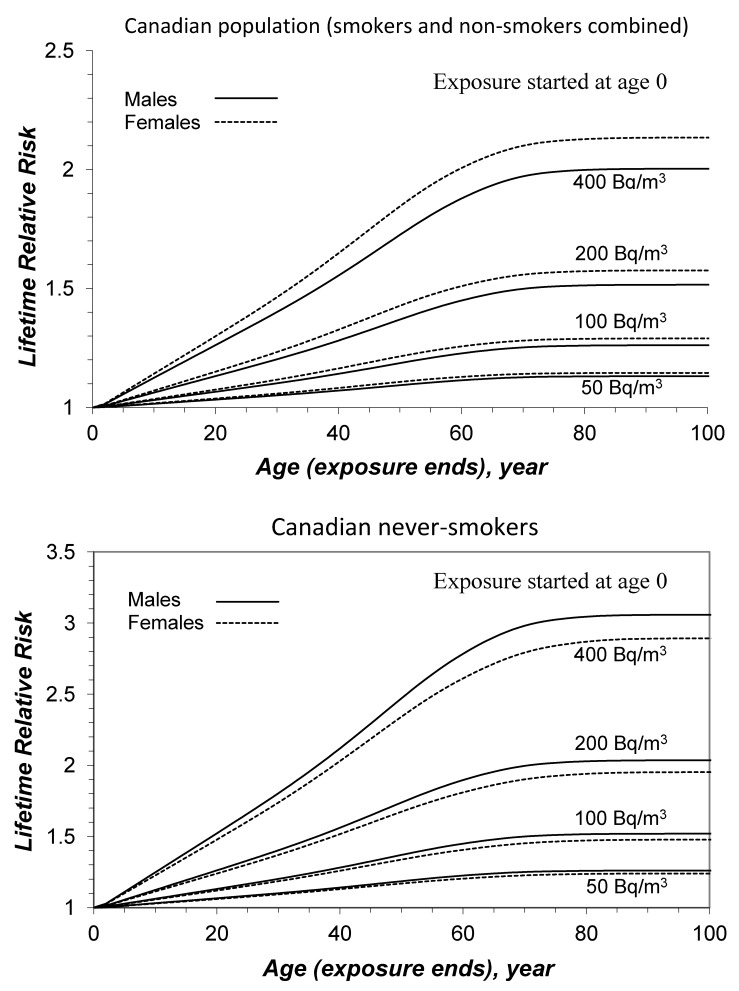
Lifetime relative risk as a function of age when exposure ends, for exposures to radon concentrations of 50, 100, 200, and 400 Bq/m^3^ (upper panel for Canadian population—A mixture of smokers and non-smokers; lower panel for male and female never-smokers).

The lifetime relative risks of lung cancer resulting from lifetime exposure to various radon concentrations were calculated for Canadian males and females, provided in Figure 1. It is well understood that the risk of developing lung cancer increases with radon concentration and exposure duration. To view the exposure duration effect more clearly, graphical examples of lifetime relative risks are given in Figure 2 for Canadian males and females, respectively. They are lifetime risks due to exposure to radon earlier in the life, *i.e.*, the exposure starts at age 0 and ends at different ages later. In all cases, the risks increase almost linearly up to age 60. Exposures after age 60 contribute very little to total lifetime risk resulting from a constant lifetime exposure. After about 36 years of exposure, both Canadian males and females reach half of their total lifetime risk for exposure to any given radon concentration. This is also true for never-smokers (lower panel of Figure 2), even though their LRR estimates are different (details on the adjustment of age-specific lung cancer mortality rates to reflect smoking status are given in a previous publication) [5].

### 1.2. LRR-Equivalency of Short Period High Exposure to Lifetime Constant Exposure

Lifetime relative risk for a shorter exposure period is of practical interest, because most people do not live in a single house for their entire life. As a result of the National Radon Program radon awareness campaign families with young children are more concerned when their homes are found to have radon concentrations significantly higher than the Canadian radon guideline of 200 Bq/m^3^. This is because exposure to radon earlier in life increases the risk of developing lung cancer during their lifetime. The Canadian radon guideline [4] recommends that remedial measures be undertaken in a dwelling whenever the average annual radon concentration exceeds 200 Bq/m³ in the normal occupancy area, and the higher the radon concentration, the sooner remedial measures should be undertaken. 

In the following calculations, it is assumed that a child (<16 years of age) lived in a house with high radon concentration for several years, and this individual lived in other houses for the rest of his/her life where radon concentrations were very low and, practically, of no real health concern. Lifetime relative risks for exposure to radon concentrations of 1,000, 2,000, 4,000, 8,000, and 10,000 Bq/m^3^ for 1, 2, and 5 years are given in Table 1 and Table 2 for males and females, respectively. For each LRR, the risk equivalent radon concentration for lifetime exposure (RERCLE) is given. For example, the LRR of developing lung cancer for a boy who lived in a house with a radon level of 2,000 Bq/m^3^ for 2 years is equivalent to a lifetime exposure to 53 Bq/m^3^.

**Table 1:** Risk equivalent radon concentration for lifetime exposure (RERCLE) when Canadian boys are exposed to radon concentrations of 1,000, 2,000, 4,000, 8,000, and 10,000 Bq/m^3^ for short periods of 1, 2 and 5 years.

**Bq/m^3^**	**1-year exposure**	**RERCLE**	**2-year exposure**	**RERCLE**	**5-year exposure**	**RERCLE**
	LRR	Bq/m^3^	LRR	Bq/m^3^	LRR	Bq/m^3^
1,000	1.03	13	1.07	26	1.17	66
2,000	1.07	26	1.14	53	1.34	132
4,000	1.14	53	1.28	105	1.67	263
8,000	1.28	105	1.54	211	2.30	527
10,000	1.34	132	1.67	263	2.60	660

**Table 2:** Risk equivalent radon concentration for lifetime exposure (RERCLE) when Canadian girls are exposed to radon concentrations of 1,000, 2,000, 4,000, 8,000, and 10,000 Bq/m^3^ for short periods of 1, 2 and 5 years.

**Bq/m^3^**	**1-year exposure**	**RERCLE**	**2-year exposure**	**RERCLE**	**5-year exposure**	**RERCLE**
	LRR	Bq/m^3^	LRR	Bq/m^3^	LRR	Bq/m^3^
1,000	1.04	14	1.08	27	1.20	68
2,000	1.08	27	1.16	54	1.39	135
4,000	1.16	54	1.31	108	1.78	271
8,000	1.31	108	1.62	217	2.52	542
10,000	1.39	135	1.78	271	2.88	677

If a child lived in a house with a radon concentration of 8,000 Bq/m^3^ for two years, the *LRR* of developing lung cancer is equivalent to a lifetime living in a house with a radon concentration of about 200 Bq/m^3^. For the same exposure conditions, lifetime relative risks are slightly higher for girls than for boys.

**Figure 3:**
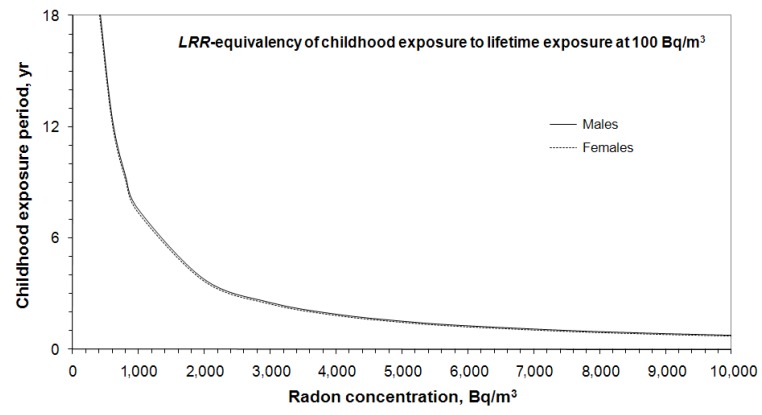
*LRR*-equivalency of short period high radon exposure in childhood to lifetime exposure to 100 Bq/m^3^.

For family safety, many parents are willing to keep their indoor radon concentrations well below 200 Bq/m^3^ guideline value, such as a level of 100 Bq/m^3^. The *LRR*-equivalency between short period high radon exposure in childhood and lifetime exposure to radon of 100 Bq/m^3^ is given in Figure 3 where *LRR* = 1.26 for males and *LRR* = 1.29 for females. If a child lived in a home with radon concentration of 2,000 Bq/m^3^ for 4 years, the *LRR* is equivalent to a lifetime exposure to radon of 100 Bq/m^3^; *i.e.*, the risk is about 30% higher than the respective baseline lung cancer risk. 

**Figure 4:**
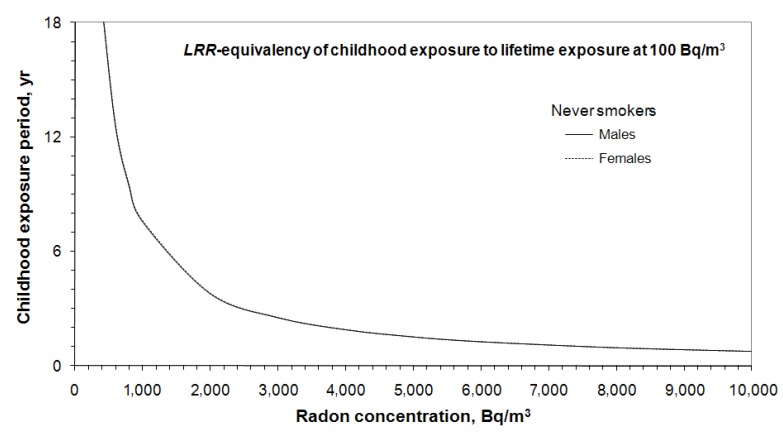
*LRR*-equivalency of short period high radon exposure in childhood to lifetime exposure to 100 Bq/m^3^ for never smokers.

The above discussion is for a general population without any adjustment made for smoking effect. The risks for a population consisting of a mixture of ever-smokers and never-smokers have values between those for ever-smokers and never-smokers. While smoking is the main cause of lung cancer among smokers, exposure to radon is the number one cause of lung cancer for most people who never smoked in their life. Assuming a child lives in a high radon home for a short period and will never smoke for the rest of his/her life, as more than 80% of Canadians do not smoke, the corresponding *LRR*-equivalency between short period exposure to high radon in childhood and lifetime exposure to radon of 100 Bq/m^3^ is given in Figure 4, where *LRR* = 1.52 for male never-smokers and *LRR* = 1.48 for female never-smokers. The *LRR*-equivalency for never-smokers given in Figure 4 is almost equivalent to the general population provided in Figure 3. However, for lifetime exposure to 100 Bq/m^3^, the *LRR* is higher for never-smokers than for a general population (*LRR* ≈ 1.5 for never-smokers *vs. LRR* ≈ 1.3 for a general population). If a child lived in a home with radon concentration of 2,000 Bq/m^3^ for four years, the *LRR* is also equivalent to lifetime exposure to radon of 100 Bq/m^3^ for individuals who will never smoke in their lifetime. In this case, the risk is about 50% higher than the baseline lung cancer risk of never smokers.

**Figure 5:**
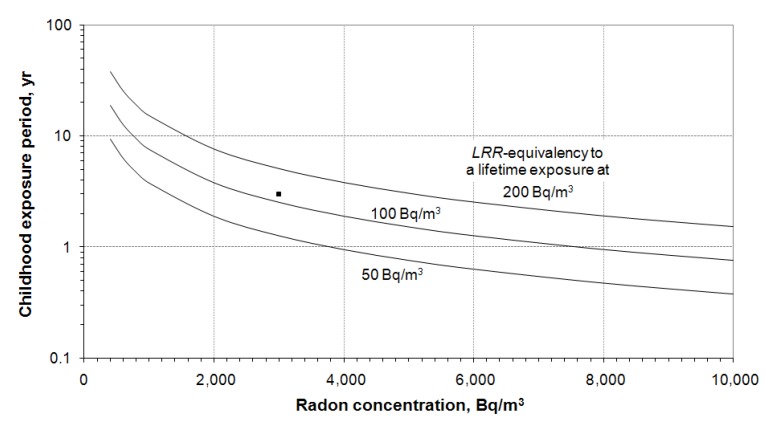
*LRR*-equivalency of short period high radon exposure in childhood to lifetime exposure to 50, 100 and 200 Bq/m^3^, respectively. The square symbol is an example of an exposure at 3,000 Bq/m^3^ for three years.

There is no significant difference in *LRR*-equivalency between males and females and between a general population and never-smokers, as shown in Figure 3, Figure 4. For practical use of radon risk equivalency, a summary is given in Figure 5. The lines are *LRR*-equivalencies to lifetime exposure to 50, 100 and 200 Bq/m^3^, respectively. Since the radon risk increases almost linearly with radon concentration and exposure duration, Figure 5 can be used to interpret other exposure situations. An example is given in Figure 5; an individual exposed at 3,000 Bq/m^3^ for 3 years (the square symbol). In this special case, the *LRR*-equivalency is about 120 Bq/m^3^ of lifetime radon exposure.

## 2. Conclusions

The risk of developing lung cancer increases with radon concentration and duration of exposure. Even though lung cancer does not normally occur in childhood, exposure to radon during childhood increases the lifetime risk of developing lung cancer, *i.e.*, the risk of developing lung cancer later in the life. The above discussion shows that if a child lived in a home with very high radon concentration for only a few years, the risk of developing lung cancer later in the life could be equivalent to a lifetime exposure to moderate radon concentration. The equivalency of lifetime risk between short period high exposure in childhood and lifetime constant exposure clearly supports the Canadian Radon Guideline recommendation that the higher the radon concentration, the sooner remedial measures should be undertaken. It should be mentioned that the risk model used in this study provides the centre estimates with scientific knowledge and data currently available. The uncertainties in risk estimates are large for the general population and even larger for the effects resulted from childhood radon exposure. 
